# 2-methoxyestradiol is effective in 2D and 3D models of NCI-H2170 lung squamous cell carcinoma cells

**DOI:** 10.1371/journal.pone.0355486

**Published:** 2026-08-04

**Authors:** Paul Mellor, Stephanie Kendall, Deborah H. Anderson

**Affiliations:** 1 Cancer Research Group, University of Saskatchewan, Saskatoon, Saskatchewan, Canada; 2 Department of Oncology, University of Saskatchewan, Saskatoon, Saskatchewan, Canada; 3 Discovery and Translational Research, Saskatchewan Cancer Agency, Saskatoon, Saskatchewan, Canada; Mayo Clinic Cancer Center, UNITED STATES OF AMERICA

## Abstract

Lung squamous cell carcinoma (LUSC) is a difficult cancer to treat, with few targeted therapies to improve its poor prognosis. The goal of this study was to use a drug repurposing strategy to evaluate and compare drug sensitivities using 2D adherent and 3D spheroid models of NCI-H2170 LUSC cells. Both 2D adherent and 3D spheroid models were used to grow NCI-H2170 lung squamous cell carcinoma cells and evaluate their sensitivity to a large library of food and drug administration (FDA)-approved drugs, including many not typically used as anti-cancer agents. Cell death was assessed in the 2D adherent models, and for the top drugs half maximal effective concentration (EC_50_) values were determined. For the 3D spheroid models, drugs reducing spheroid size after 4 days of treatment were identified. There were 263 drugs that reduced the cell viability to <20% when cells were grown in 2D in 10 µM drug. When grown in 3D the cells were generally more drug resistant, with 87 drugs capable of reducing spheroid volume when grown over 4 days in 10 µM drug. Interestingly, 60 drugs proved effective in both model systems including many drugs that typically associated with anti-cancer properties. Of these 60, four were further found to have selective effects towards metastatic NCI-H2170 cells as compared to a much less metastatic matched cell line expressing the metastasis suppressor CREB3L1, in both 2D and 3D model systems. These included the hypoxia-inducible factor 1-alpha (HIF-1α inhibitor 2-methoxyestradiol, and three anti-infection agents (cetylpyridinium chloride, chlorhexidine-2HCl, zinc pyrithione).

**Conclusions:**

These results suggest that the HIF-1α inhibitor 2-methoxyestradiol, and three anti-infection agents (cetylpyridinium chloride, chlorhexidine-2HCl, zinc pyrithione) may be effective for metastatic LUSCs.

## Introduction

Lung cancer is the most diagnosed cancer worldwide (11.6% of all cancer types) and the leading cause of death due to cancer (18.4% of all cancers) [1]. There are large variations in the rates of lung cancer across the globe, in part reflecting the use and type of cigarettes smoked [1]. Lung cancers can be divided into non-small cell lung cancer (NSCLC, 85%) and small cell lung cancer (15%), with NSCLCs consisting of subtypes lung adenocarcinoma (LUAD, 37–45%), lung squamous cell carcinoma (LUSC, 22–33%) and large cell carcinoma (4–9%) [2]. LUSC is most often linked to a history of smoking, whereas adenocarcinoma occurs in smokers, former smokers and non-smokers.

Several targeted therapies have been used to treat subpopulations of lung cancer patients including immunotherapy (e.g., Nivolumab, Atezolizumab), angiogenesis inhibitors (e.g., Avastin), epidermal growth factor receptor (EGFR) inhibitors (e.g., Erlotinib, Iressa, Osimertinib-for EGFR-M790M) and ALK inhibitors (e.g., Crizotinib; 5% of NSCLCs with ALK rearrangements) [3]. While these targeted therapies have shown some promising results, many patients are not candidates for these drugs and those that are, frequently develop resistance to these treatments. There are few if any effective targeted therapies for LUSC, which typically do not contain the molecular features targeted by these agents [4,5]. As such, LUSCs do not respond well to therapy resulting in a 5-year survival rate of only 16% [6,7].

The main treatment options for LUSC are surgery, chemotherapy and radiation therapy. The Cancer Genome Atlas reported a large-scale genomic analysis of LUSCs, identifying alterations in genes involved in oxidative stress pathways and differentiation, including *NFE2L2, KEAP1, CUL3, SOX2, TP63, NOTCH1, NOTCH2, ASCL4* and *FOXP1* [5]. Gene expression analysis also revealed alterations in the PI3K pathway (*PTEN, PIK3CA*), receptor tyrosine kinase signaling (*FGFRs, ERBs, JAKs, BRAF*) as well as cell cycle regulation pathways (*TP53, CDKN2A, RB1*). It remains to be seen, if one or more of these molecular changes are effective targetable vulnerabilities for the subpopulation of LUSCs containing them.

Our bioinformatics analyses of large tumor datasets highlighted a unique feature of most LUSC cases, a highly significant (p = 1.7x10^-19^) reduction in CREB3L1 (cAMP responsive element binding protein 3 like 1) expression [8]. This reduction in CREB3L1 expression was not observed in LUAD which expressed similar levels to normal lung tissue, suggesting that these subtypes of lung cancer are molecularly distinct. CREB3L1 has previously been shown to suppress the metastatic properties in cancers of the breast, bladder and ovarian cancer [8–11]. More recently, we have shown that CREB3L1 also functions as a metastasis suppressor in LUSCs, suggesting a possible role for CREB3L1 loss in the aggressive nature of LUSC [12].

CREB3L1 is a transcription factor that is initially localized to the endoplasmic reticulum. In response to cell stress, CREB3L1 traffics to the Golgi complex where it is cleaved by two site-specific proteases, to liberate a soluble active transcription factor, which translocates into the nucleus to regulate the expression of genes [13–17]. Cancer cells experience considerable stress from poorly folded proteins (resulting from oncogenic mutations), as well as a poor supply of nutrients and oxygen as the tumors grow away from the blood supply [18]. Stress response proteins can reduce the impact of these factors by increasing the expression of protein folding chaperones, and by decreasing protein translation in the endoplasmic reticulum [19–24]. CREB3L1 is a unique stress response protein, which represses the expression of genes that promote cell growth and cell survival, as well as cell migration, invasion, angiogenesis and metastasis [9,10,12]

The majority of LUSC tumors have lost expression of CREB3L1, and re-expression of CREB3L1 in these CREB3L1-low LUSC cells reduces cell migration, invasion and anchorage-independent growth in soft agar [12]. CREB3L1 also represses tumor formation and blocks metastasis in mouse xenograft models [12]. Differential gene expression analysis comparing LUSC and CREB3L1-re-expressing LUSC cells strongly supports a role for CREB3L1 in repressing a large number of signaling pathways that contribute to tumor progression, cell migration, invasion, angiogenesis and metastasis [12]. These results indicated that CREB3L1 functions as a metastasis suppressor in LUSC. The objective of this work was to identify new and effective drugs for LUSCs using a drug repurposing approach and to compare the results side-by-side in 2D adherent versus 3D spheroid models. As part of this work, we also set out to identify drugs that are specifically more effective against the more metastatic LUSCs (CREB3L1-low) as compared to the same cells expressing CREB3L1 which are less metastatic. Several drugs were newly identified as having anti-cancer activity, effective in 2D and 3D models of LUSC. Some drugs also showed higher efficacy towards more metastatic LUSC cells. This report identified several promising new drugs to evaluate in future preclinical and clinical studies of LUSC.

## Materials and methods

### Cell lines and cell culture

The lung squamous cell carcinoma cell line NCI-H2170 was obtained from the American Type Culture Collection (ATCC, Gaithersburg, MD). Cells were authenticated by the supplier (http://www.ATCC.org) and cultured as recommended by ATCC for less than six months from the time of resuscitation. NCI-H2170 cells stably expressing triple hemagglutinin (HA)-tagged CREB3L1 (HA-CREB3L1) have been described and characterized previously with both cell lines having similar plating efficiencies and doubling times [12].

### Primary drug screen for adherent cells (2D)

A high-throughput drug screen of an FDA-approved drug library (2,580 compounds; Selleckchem, Houston, TX, USA, L1300) was carried out on NCI-H2170 cells. Briefly, NCI-H2170 cells (2x10^3^) were seeded into 384-well black-walled plates (142761, NUNC) in a total volume of 50 µL/well in RPMI-1640 + 10% fetal bovine serum. Cells were allowed to attach and grow at 37˚C and 5% CO_2_. Most drugs were provided as 10 mM stocks in dimethyl sulfoxide (DMSO) or water and were diluted 200-fold in media to give 50 µM (5x final desired). There were also a small number of drugs provided as 2 mM stocks in either DMSO or water. These were diluted 40-fold in media to give 50 µM (5x final desired). Prior to adding drugs to cells, 10 µL of media was removed from each well of cells. Drugs were added to each well (10 µL; 50 µM) giving a final concentration of 10 µM using an ASSIST PLUS pipetting robot and a 16-channel VIAFLO pipette (4505 and 4642, INTEGRA Biosciences AG, Hudson, NH). Control DMSO wells were also included to control for impacts independent of the test drug. After 4 days of drug treatment cells were stained with Hoechst and ImageIT Dead Green to quantify total and live cells. Cell viability was determined by quantifying total cells and dead cells as previously described [25]. Briefly, cells were stained in media containing Hoechst 33342 dye (5 µM; ThermoFisher Scientific, Saskatoon, SK, 62249) and ImageIT Dead Green dye (100 nM; ThermoFisher Scientific, Saskatoon, SK, I10291) and imaged using a Thermo Scientific^TM^ CellInsight^TM^ CX7 High Content Screening (HCS) Platform. Images were analyzed using Thermo Scientific^TM^ HCS Studio 3 Cell Analysis Software to quantify total cells (Hoechst-stained nuclei) and dead cells (ImageIT Dead Green). The total live cell counts for each well containing a drug was normalized to the total live cell count for each control well to account for any dead cells that had lifted off the plate in test wells. The test well total live cell count was divided by the average of the corresponding solvent control total live cell count, giving the percentage cell viability (% viability). The mean ± standard error of the mean (SEM) of the % viability of each set of triplicate wells was determined.

### Secondary drug screen for adherent cells and EC_50_ determinations

Drugs selected from the primary screens [60] were validated for their effects and EC_50_ values (concentration of compound that gives half-maximal response) determined over a range of drug concentrations using both NCI-H2170 and NCI-H2170 + HA-CREB3L1 cells (similar doubling times). Typically, drugs were serially diluted 1:3 in media (e.g., 0–10 µM), using the appropriate solvent control wells. Graphs and EC_50_ values were generated using PRISM software (GraphPad, San Diego, CA, v9.2.0) using a non-linear curve fit from triplicate measurements.

### Primary and secondary drug screen for spheroids (3D)

Three-dimensional spheroids of NCI-H2170 cells were formed by seeding 2x10^3^ cells/well in Ultra-low adhesion 96 well plates (Sigma-Aldrich, Corning Costar 7007) in a volume of 200 µL of RPMI-1640 + 10% fetal bovine serum. A single spheroid formed in each well over a 5-day period, prior to the addition of test drugs. Drugs were diluted to 50 µM (5x final desired) as described above. To add drugs 40 µL of media was carefully removed from each spheroid-containing well, followed by the addition of 40 µl of 5x drug to give a final drug concentration of 10 µM. After 4 days of drug treatment, spheroid diameters (d) were measured using the Thermo Scientific^TM^ CellInsight^TM^ CX7 High Content Screening (HCS) Platform using brightfield settings. The volume of each spheroid was calculated (4/3*π*(d/2)^3^) and the % volume was defined relative to the solvent control spheroid (100%). The mean ± standard error of the mean (SEM) of the % volume of each set of triplicate wells was determined.

Secondary validation experiments were carried out similarly except using a final drug concentration of 1 µM. The volumes reported were defined relative to the DMSO control (100%). The mean ± SEM from 4 spheroids from each condition was reported.

Selected drugs [60] were evaluated for their differential effects using both NCI-H2170 and NCI-H2170 + HA-CREB3L1 cells (less metastatic than parental NCI-H2170 [12]) in 3D at 10 µM similarly. Volumes for drug treated spheroids were reported relative to solvent control spheroids (100%). The mean ± SEM from 4 biological replicates from each condition.

### Statistical analysis

Statistically significant differences upon drug treatment as compared to the DMSO control were determined from three independent experiments using two-tailed one-sample t-tests, and p-values were adjusted using the Benjamini-Hochberg false discovery rate (FDR) procedure using PRISM software (GraphPad, San Diego, CA, v9.2.0), significance considered at the desired FDR Q value < 0.05 (the adjusted p-value).

## Results

### High-throughput drug screens to identify compounds that are cytotoxic towards NCI-H2170 LUSCs

**Fig 1:**
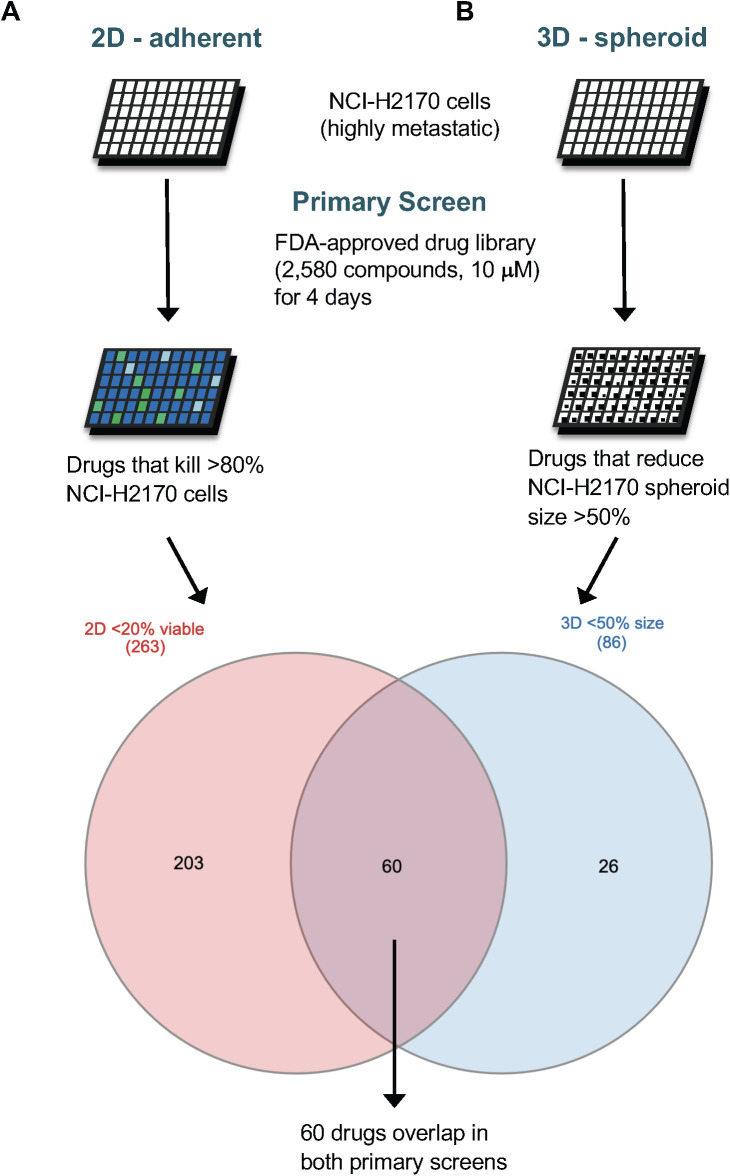
Schematic diagram of primary drug screens. NCI-H2170 LUSC cells were evaluated in for drug sensitivity using an FDA-approved drug library of 2,580 compounds in both a 2D adherent cell culture model system (A) and a 3D spheroid model system (B). Cells were treated with 10 µM drug for 4 days and evaluated for cell viability/death in the 2 D model and spheroid size reduction in the spheroid model. There were 60 drugs in common that caused both a reduction in viability of <20% for adherent cells (263) and also reduction the spheroid size >50% for NCI-H2170 cells (86).

To identify drugs that are cytotoxic towards NCI-H2170 LUSC cells, we screened a drug library consisting of 2580 FDA-approved compounds (S1 Table) using two independent screens carried out in parallel. The first screen evaluated the cytotoxicity of each drug towards NCI-H2170 LUSCs grown in standard 2D adherent culture conditions (10 µM, 4 days). To quantify total cells remaining in each well, cells were stained with Hoechst 33342 dye, and to quantify apoptotic cells, ImageIT Dead Green was used and compared to vehicle-treated control wells to obtain % viability. To rigorously detect true pharmacological hits while limiting false positive discoveries from multiple testing, a Benjamini-Hochberg false discovery rate (FDR) adjustment (Q < 0.05)) was applied to the underlying one-Sample t-tests. There were 263 drugs that reduced the cell viability of the NCI-H2170 LUSCs to <20% (Fig 1A, S2 Table).

Since spheroid model systems have been suggested to better recapitulate the complexity of patient tumors [26–29], including nutrient and hypoxic gradients, 3D cell-cell and cell-matrix interactions, and demonstrate resistance to drug treatment [26–29], a second screen was carried out on NCI-H2170 cells grown as spheroids. The diameters of the spheroids were assessed after 4 days of drug treatment (10 µM) and the volume of each was calculated and compared to vehicle control spheroids.

There were 87 drugs that reduced spheroid volumes of the NCI-H2170 LUSCs to <50%, with reductions in spheroid volume resulting from drug impacts on cell growth and/or cell viability (Fig 1B, S3 Table). This analysis does not distinguish between these two possibilities. To focus on a manageable number of the top hits appearing in both the 2D and 3D screen, we selected cut-offs for each screen. There were 60 drugs that both induced cell death (<20% viability; 2D results) and reduced growth of 3D spheroids (>50% reduction in sphere volume) that were selected for further analysis (Fig 1, Table 1). About half of these drugs are known to inhibit well known cancer targets including tyrosine kinsases (Abl, Src, c-Kit, EGFR, human epidermal growth factor receptor 2 (HER2), Btk, PDGFR, VEGFR, Syk), and topoisomerases. Interestingly, about one-third of these drugs showed previously unappreciated anti-cancer activity, with their major uses as anti-infection agents, or a broad variety of other functions. These results suggest that this latter group may serve as novel anti-cancer agents worthy of future investigation.

**Table 1:** Drugs both capable of blocking NCI-H2170 3D spheroid growth (>50% reduction) and cytotoxic towards NCI-H2170 cells grown in 2D (>80% killing).

Drug Name	NCI-H2170 3D spheroid volume compared to vehicle treated controls (%)	NCI-H2170 2D viability compared to vehicle treated controls (%)	Target	Pathway
Avermectin B1 (Abamectin)	29.79	11.83	Anti-infection	Microbiology
Piroctone Olamine	42.55	16.72	Anti-infection	Microbiology
Rotenone (Barbasco)	46.33	2.89	Anti-infection	Microbiology
Cetylpyridinium Chloride	49.25	0.62	Anti-infection	Microbiology
Buparvaquone	36.72	10.38	Anti-infection	Microbiology
Proflavine Hemisulfate	37.94	3.97	Anti-infection	Microbiology
Proflavine	40.20	4.72	Anti-infection	Microbiology
Chlorhexidine diacetate	43.88	3.28	Anti-infection	Microbiology
Chlorhexidine-2HCl	46.35	6.65	Anti-infection	Microbiology
Ciclopirox	30.48	18.97	ATPase,Anti-infection	Transmembrane Transporters
Ciclopirox ethanolamine	34.28	15.54	ATPase	Transmembrane Transporters
Chlorhexidine	38.61	5.19	Bacterial	Infection
Dasatinib hydrochloride	24.71	11.57	Abl/Src	Kinase
Dasatinib	47.07	4.25	Bcr-Abl,c-Kit,Src	Angiogenesis
Dasatinib Monohydrate	28.39	10.14	Bcr-Abl,c-Kit,Src	Angiogenesis
Ivermectin	41.29	1.80	Chloride Channel,Anti-infection	Transmembrane Transporters
Triapine	38.07	9.86	DNA/RNA Synthesis	DNA Damage
Erlotinib	31.61	19.83	EGFR	Protein Tyrosine Kinase
Lapatinib ditosylate monohydrate	31.53	11.12	EGFR,HER2	Protein Tyrosine Kinase
Neratinib (HKI-272)	44.33	7.43	EGFR,HER2	Protein Tyrosine Kinase
Olmutinib (HM61713, BI 1482694)	45.58	2.45	EGFR,BTK	Protein Tyrosine Kinase
Simeprevir	48.68	5.23	HCV Protease	Proteases
Tucidinostat (Chidamide)	47.28	11.05	HDAC	Epigenetics
2-Methoxyestradiol (2-MeOE2)	46.60	10.16	HIF	Angiogenesis
Nelfinavir Mesylate	42.97	5.95	HIV Protease	Proteases
Tanshinone IIA	22.46	5.92	Lipase	Metabolism
Zotarolimus (ABT-578)	31.64	18.55	mTOR	PI3K/Akt/mTOR
Temsirolimus (CCI-779, NSC 683864)	48.11	11.75	mTOR	PI3K/Akt/mTOR
Triptolide (PG490)	30.49	11.85	NF-κB	NF-κB
Phenazine methosulfate	30.43	12.52	Others	Others
Lomitapide Mesylate	42.13	2.42	Others	Others
Quinacrine Dihydrochloride Dihydrate	42.61	7.30	Others	Others
Ciclesonide	44.18	9.40	Others	Others
Triptonide	47.20	7.78	Others	Others
Fenretinide	49.57	1.30	Others	Others
Ethidium bromide	30.43	0.88	Others	Others
1, 10-Phenanthroline monohydrate	30.45	14.30	Others	Others
Doramectin	38.80	5.53	Others	Others
Meclocycline Sulfosalicylate	48.42	14.36	Others	Others
Deferasirox	48.77	7.22	P450 (e.g., CYP17)	Proteases
Niraparib (MK-4827) tosylate	47.85	18.20	PARP	DNA Damage
Sunitinib	43.42	6.88	PDGFR,c-Kit,VEGFR	Protein Tyrosine Kinase
Sorafenib Tosylate	45.67	12.73	PDGFR,Raf,VEGFR	MAPK
Regorafenib Monohydrate	49.48	9.38	PDGFR,VEGFR,c-Kit,c-RET,Raf	Protein Tyrosine Kinase
Pentamidine isethionate	48.95	15.76	phosphatase	Others
Cyclosporine	34.52	15.24	phosphatase,Immunology & Inflammation related	Others
Hexachlorophene	30.32	12.69	Potassium Channel	Transmembrane Transporters
Zinc Pyrithione	37.18	0.39	Proton Pump,Anti-infection	Transmembrane Transporters
Fostamatinib (R788)	39.49	14.78	Syk	Angiogenesis
Eltrombopag Olamine	48.29	9.98	Thrombin	Others
Mitoxantrone 2HCl	31.91	6.42	Topoisomerase	DNA Damage
Hydroxy Camptothecine	34.86	9.84	Topoisomerase	DNA Damage
Nitroxoline	38.00	6.82	Topoisomerase	DNA Damage
Amonafide	38.61	6.54	Topoisomerase	DNA Damage
(S)-10-Hydroxycamptothecin	41.26	12.85	Topoisomerase	DNA Damage
Daunorubicin HCl	41.45	2.12	Topoisomerase	DNA Damage
Epirubicin HCl	48.45	1.70	Topoisomerase	DNA Damage
Camptothecin	49.49	19.80	Topoisomerase	DNA Damage
Mozavaptan	48.88	18.59	Vasopressin Receptor	GPCR & G Protein
Salinomycin (from Streptomyces albus)	49.31	19.53	Wnt/beta-catenin	Stem Cells & Wnt

### Differential drug sensitivity in NCI-H2170 (metastatic) compared to NCI-H2170 + HA-CREB3L1 (less metastatic) LUSCs

For these 60 overlapping drug hits, we carried out a validation and EC_50_ analysis using 2D adherent cells. To highlight drugs which were more effective against the NCI-H2170 metastatic cells, we carried out a parallel analysis using less metastatic NCI-H2170 + HA-CREB3L1 cells [12]. Using the paired cell lines with different metastatic potential enabled us to identify drugs that affected the cells similarly, and also those that were preferentially cytotoxic towards the more metastatic NCI-H2170 cells.

**Fig 2:**
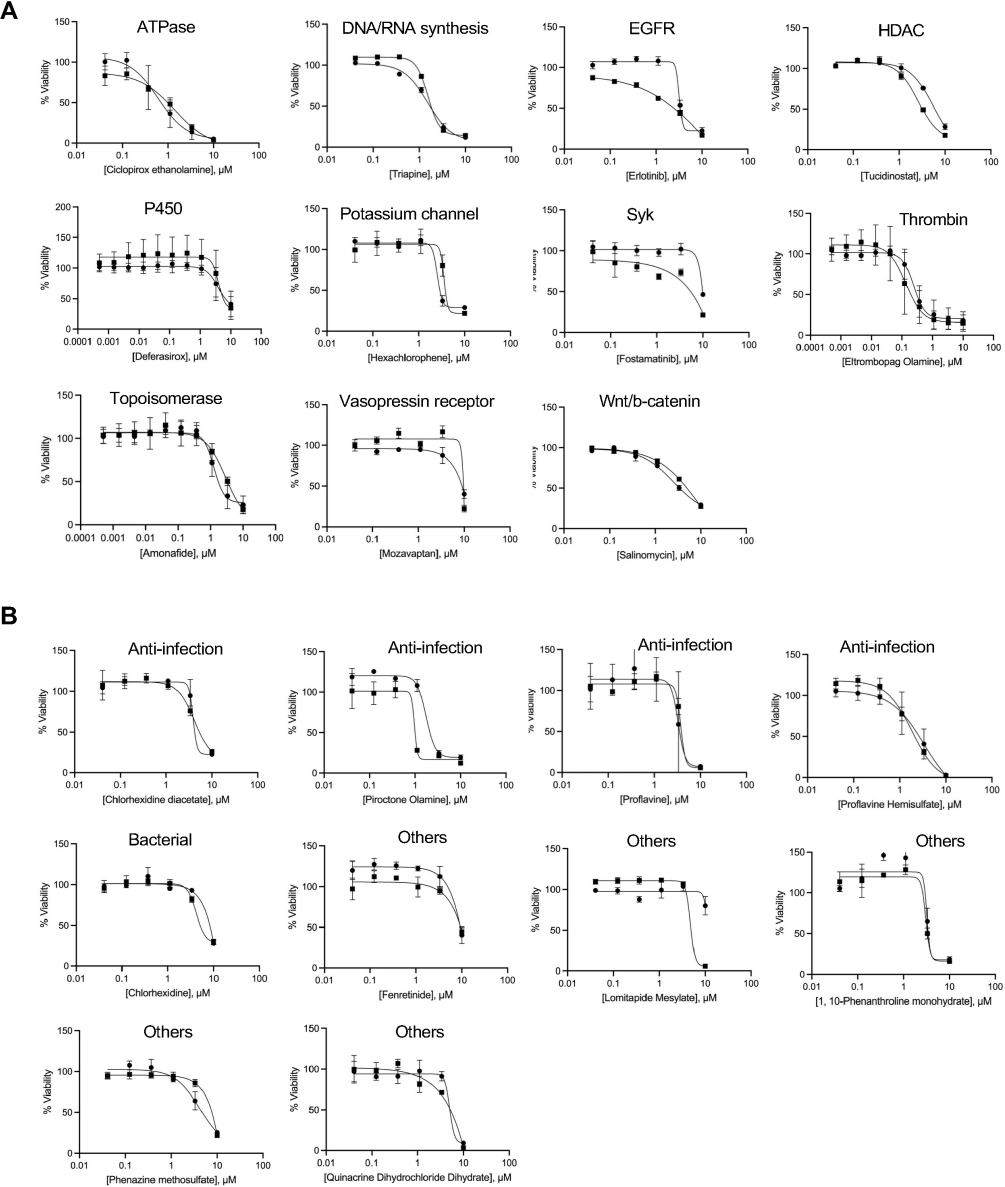
Drugs effective at killing 2D NCI-H2170 and NCI-H2170 + HA-CREB3L1 LUSC cells. NCI-H2170 (circles; metastatic) and NCI-H2170 + HA-CREB3L1 LUSC (squares; poorly metastatic) cells were treated with the indicated drugs over a range of concentrations up to 10 µM as detailed in the methods. The number of live cells in the drug treated samples was quantified and compared to total cells in vehicle control wells to determine cell viability at each drug concentration. Drugs with known specific targets (**A**) and those with less well-defined targets (**B**) are shown.

Fourteen of the 60 drugs evaluated were highly cytotoxic to both cell lines and included most topoisomerase inhibitors, as well as several inhibitors of kinases (EGFR, HER2, Bcr-Abl, c-Kit, Src) and nuclear factor kappa-light-chain-enhancer of activated B cells (NFkB) (S1 Fig). Three drugs had little or no effect on both cell lines, proving ineffective, including two mTOR inhibitors (S2A Fig) and another 17 drugs with diverse targets were somewhat effective but at high concentrations (>10 µM; S2B Fig). Twenty-one drugs were effective at reducing cell viability to a similar extent in both cell lines with EC_50_ values <10 µM, some with known targets (Fig 2A), some of which are known to have anti-cancer properties. Other drugs equally effective in both cell lines have not typically been associated with anti-cancer roles (Fig 2B). These results suggest that there are a number of drugs with novel anti-cancer activity including anti-infection agents (chlorhexidine diacetate, piroctone olamine, proflavine, proflavine hemisulfate), bacterial agents (chlorhexidine), and others with less well described functions (fenretinide, lomitapide mesylate, 1,10-phenanthroline monohydrate, phenazine methosulfate, quinacrine dihydrochloride dihydrate).

**Fig 3:**
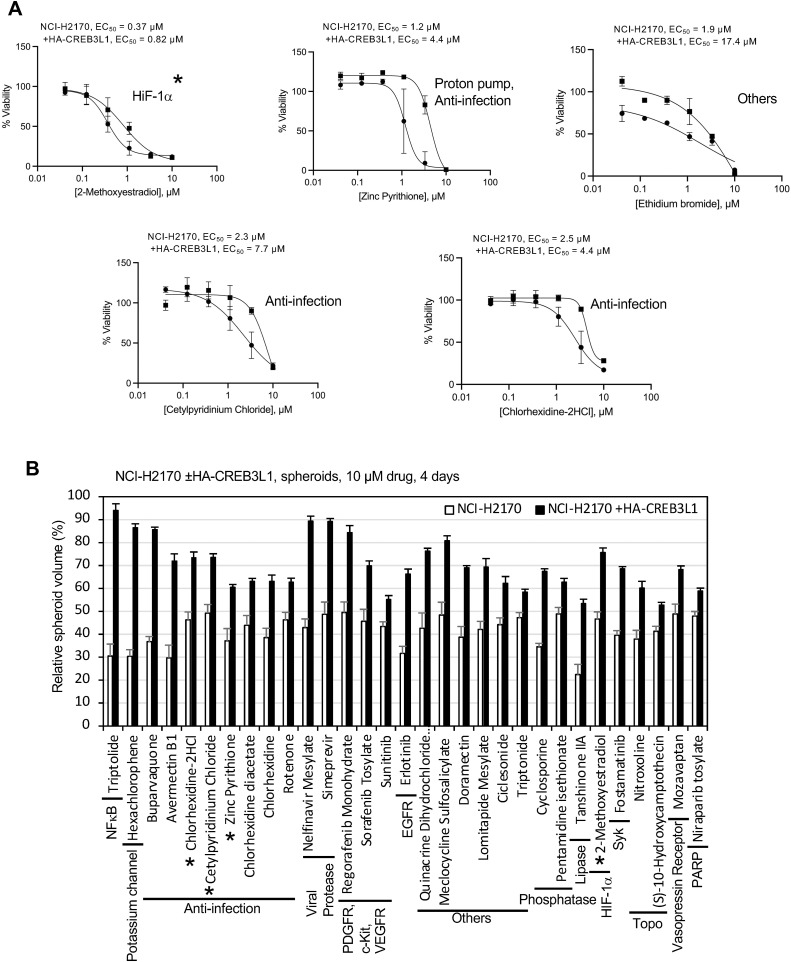
Drugs more effective against metastatic NCI-H2170 LUSC cells as compared to their less metastatic CREB3L1-expressing counterparts. **A**) The indicated cells were grown in 2D and treated with drug as detailed in Fig 2, and showed enhanced killing of metastatic NCI-H2170 cells as compared to NCI-H2170 + HA-CREB3L1 cells with lower EC_50_ values. **B**) The indicated cells were grown as 3D spheroids and treated with drug (10 µM) for 4 days. The volume of each spheroid was measured to quantify the impact of drug treatment compared to the DMSO control treated samples. Only drugs showing significant differences are shown here (remaining data in S3 Fig). The mean ± SEM from 4 biological replicates from each condition.

There were 5 drugs that reduced cell viability in both cell lines which also showed preferential cytotoxicity towards the more metastatic NCI-H2170 cells (Fig 3A). These included the HiF-1α inhibitor 2-methoxyestradiol, the DNA intercalating agent ethidium bromide, and several drugs best known as anti-infection agents (zinc pyrithione, cetylpyridinium chloride, chlorhexidine-2HCl). These results suggest that some drugs may be more effective at killing metastatic cells.

To identify drugs more effective towards metastatic LUSCs in a 3D spheroid model, we evaluated the 60 drugs (10 µM) side by side in NCI-H2170 cells (more metastatic) as compared to NCI-H2170 + HA-CREB3L1 (less metastatic) [12]. These 60 drugs caused at least a 50% reduction in NCI-H2170 spheroid size, compared to treatment with vehicle control (Table 1, Fig 3B). Interestingly, drug treatments of the NCI-H2170 + HA-CREB3L1 spheroids showed a range of effects (Fig 3B; S3A Fig). Some drugs caused only small reductions in spheroid size (Fig 3B), whereas other drugs showed >50% reductions as seen for NCI-H2170 spheroids (S3B Fig), and still other drugs were most effective at decreasing NCI-H2170 + HA-CREB3L1 spheroid size (S3 Fig). The drugs most selective for metastatic cells (NCI-H2170) showed the largest differential effect between the two cell lines and included inhibitors of NFkB (triptolide), potassium channels (hexachlorophene), viral proteases (nelfinavir mesylate, simeprevir), kinases (regorafenib monohydrate, sorafenib tosylate, sunitinib, erlotinib) phosphatases (cyclosporine, pentamidine isethionate), lipases (tanshinone IIA), as well as a large number of anti-infection or drugs with poorly described functions (others) (Fig 3B). Of note, there were four drugs that showed a stronger effect towards metastatic NCI-H2170 cells as compared to less metastatic NCI-H2170 + HA-CREB3L1 cells in both the 2D and 3D model systems, the hypoxia-inducible factor 1-alpha inhibitor 2-methoxyestradiol, and three anti-infection agents (cetylpyridinium chloride, chlorhexidine-2HCl, zinc pyrithione) (Fig 3A-3B). This suggests that these drugs may be more effective in treatment of metastatic LUSCs.

### Drugs most effective in 3D spheroid models

**Fig 4:**
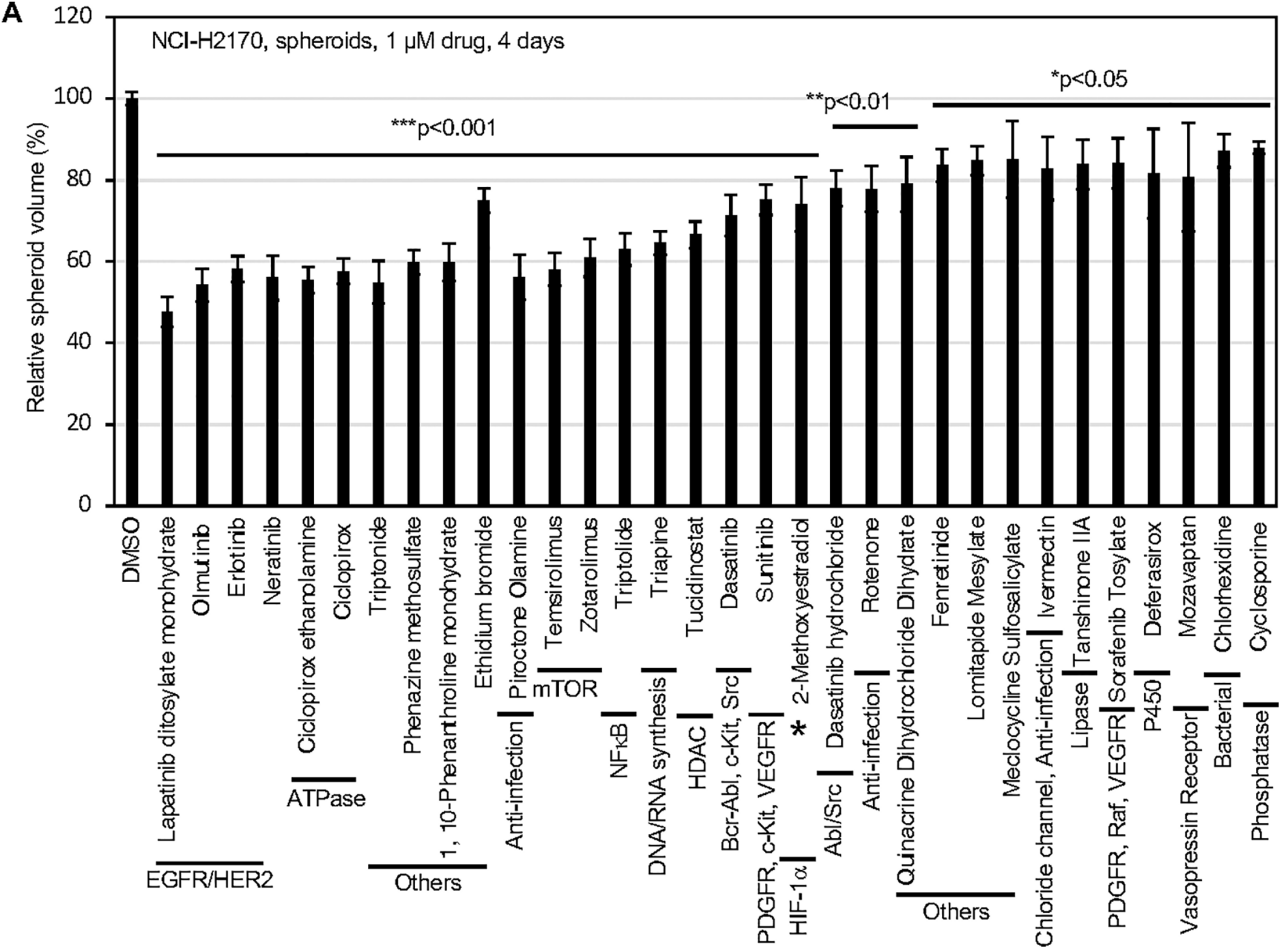
Reduced drug concentrations (1 µM) are still effective against NCI-H2170 cells grown in 3D spheroids. **A)** NCI-H2170 cells were grown as 3D spheroids and treated with drug (1 µM) for 4 days. The volume of each spheroid was measured to quantify the impact of drug treatment compared to the DMSO control treated samples. Mean ±SEM from 4 independent spheroids. A t-test was used to assess statistically significant differences. ***p < 0.001, **p < 0.01, *p < 0.05. Only drugs showing significant differences are shown here (remaining data in S4 Fig).

Lastly, we carried out a more rigorous analysis of 52 of the 60 overlapping drug hits, where we excluded the 8 topoisomerases which tend to be very cytotoxic with considerable side effects for patients. Whereas the initial 3D organoid drug screen was carried out using 10 µM drug, in this analysis we evaluated the impact of 1 µM drug treatment over 4 days for the NCI-H2170 as compared to the DMSO control. Twenty drugs did not have significant impacts on spheroid size at the 1 µM drug concentration (S4 Fig). There were 32 drugs that caused significant reductions in spheroid volume (Fig 4A). Of the four drugs showing preferential effects against more metastatic cells noted above, the HIF-1α inhibitor 2-methoxyestradiol also caused significant reductions in spheroid size when used at 1 µM. The largest and most significant reductions in spheroid size were observed for drugs targeting the EGFR/HER2 (Fig 4A). Although NCI-H2170 cells lack common activating EGFR mutations, they are considered HER2-addicted [30], consistent with our drug sensitivity findings and validating the use of this spheroid model system to identify other drugs which could be repurposed for the treatment of LUSC.

## Discussion

In this study we used a drug repurposing strategy to identify several novel drugs that may provide therapeutic benefit in the treatment of LUSC using both 2D adherent and 3D spheroid model systems. The 2D model system provided greater efficiency to carry out high-throughput drug screening. Importantly, 3D model systems are known to exhibit higher resistance to drug treatments compared to 2D monolayers due to their reduced proliferation, cell-cell interactions and architecture which includes nutrient and oxygen gradients [31,32]. As such drug sensitivity data from 3D models has been shown to predict in vivo effectiveness more accurately [31,32]. We further had the benefit of using a matched pair of well characterized LUSC cell lines (NCI-H2170 ± HA-CREB3L1; [12]) with very different metastatic properties allowing us to identify new drugs more selective towards metastatic LUSC cells. Four drugs were found to be both very effective and selective for metastatic LUSC cells, killing cells in 2D cultures and reducing 3D spheroid formation, including cetylpyridinium chloride, chlorhexidine-2HCl, zinc pyrithione and 2-methoxyestradioal.

Cetylpyridinium chloride is best known for its antiseptic properties when used in mouthwash [33], though it is now known to also exhibit anti-cancer activity through multiple pathways. It has been shown to selectively inhibit mitochondrial complex I, disrupting mitochondrial respiration and blocking ATP synthesis [34]. This activates the AMP-activated protein kinase (AMPK) pathway, an energy-sensing system, which halts ATP-consuming processes such as cell survival, proliferation and migration when they are not critical for cell survival [34,35]. Cetylpyridinium chloride has also been shown to cause endoplasmic reticulum stress and an accumulation of misfolded proteins [36]. This stimulates the ERN1-MAP3K5-p38 MAPK signaling pathway, resulting in paraptosis (a form of programmed cell death distinct from apoptosis) [36]. There are also several studies demonstrating the anti-tumor activity of cetylpyridinium chloride using in vivo mouse models of pancreatic cancer [36] and liver cancer [37].

Chlorhexidine-2HCl is another antiseptic compound with much less known role about its emerging anti-cancer activity or mechanism(s) of action [38]. It has been shown to inhibit cell proliferation, reduce colony formation in soft agar and inhibit cell migration in a wound healing assay [39]. Treatment with chlorhexidine-2HCl induces apoptosis via cleavage of caspase 3 and PARP-1 and inhibits the growth of tumor xenografts in colorectal models [39,40].

Zinc pyrithione has typically been used as an anti-fungal and anti-bacterial agent in dandruff shampoos that has shown promise as an anti-cancer agent through drug repurposing studies [41,42]. Zinc pyrithione is a deubiquitinase inhibitor, which induces the accumulation of ubiquitin-conjugated proteins, inhibiting protein degradation and causing proteasome stress and apoptosis [42]. Zinc pyrithione also enhances the uptake of extracellular zinc into cells, causing oxidative stress, mitochondrial toxicity and caspase 3-mediated apoptosis [43,44]. Treatment with zinc pyrithione has also been shown to disrupt the PI3K/AKT/mTOR pathway via reduced Akt activation, in combination with decreased expression of mTOR, Raptor, Rictor and GbL [41]. Further, zinc pyrithione results in reduced expression of Wnt/β-catenin signaling pathway components including its upstream regulator DKK3, β-catenin, its interaction partners TCF1 and LEF1, as well as their downstream targets cyclin D1 and c-Myc [41,44–46]. Zinc pyrithione is cytotoxic to several types of cancer including lung adenocarcinoma, breast cancer and prostate cancer through its ability to perturb cancer-specific zinc homeostasis [47–49].

2-Methoxyestradiol is a metabolite of 17β-estradiol that does not bind to estrogen receptors but instead functions to block angiogenesis as well as cell proliferation and promotes apoptosis [50,51]. It binds to β-tubulin disrupting microtubules, resulting in the downregulation of HIF-1α to prevent tumor survival under hypoxic conditions and suppressing angiogenesis [52,53]. Since 2-methoxyestradiol binding to β-tubulin inhibits polymerization, it causes mitotic arrest and leads to apoptosis [53,54]. In preclinical and clinical trials 2-methyloxyestradiol is effective for advanced breast, prostate, ovarian, pancreatic cancers and multiple myeloma [51,55,56]. 2-Methoxyestradiol has been shown to have anti-tumor activity in lung adenocarcinoma cells (A549) and large cell lung cancer cells (H460) [57]. Our results suggest it may be effective in the treatment of lung squamous cell carcinomas.

Our screening strategy successfully utilized the translational advantages of 3D spheroids over 2D monolayers to identify candidate drugs with established anti-cancer molecular pathways, though several constraints inherent to early-stage drug repurposing must be acknowledged. First, although the 3D spheroids capture architectural features as well as nutrient gradients that better predict clinical effectiveness than 2D systems, they still lack critical components of the tumor microenvironment (TME), including stromal cells, immune cells, and a functional vasculature. Because these isolated cultures cannot replicate systemic drug metabolism or host immune dynamics, the robust cell-killing efficacy observed in vitro may not translate to identical in vivo safety or efficacy. Furthermore, our evaluation of impact in 3D cultures relied primarily on tracking changes in spheroid size. While the prioritized candidates demonstrated definitive cytotoxicity in our parallel 2D monolayer screen, monitoring 3D volume does not distinguish between direct cell killing and cytostatic growth inhibition, both of which would be beneficial. Future work should include direct apoptotic or cell-death validation which was deferred during this 3D profiling stage to optimize screening throughput [58–60].

Future work should also include determining the clinical viability and pharmacokinetics of the specific biological hits identified, particularly the antimicrobial agents cetylpyridinium chloride, chlorhexidine-2HCl, and zinc pyrithione. Although our results and existing literature confirm their potent anti-cancer activity through a variety of mechanisms, these compounds are traditionally used for localized or topical formulations like mouthwashes, antiseptics, and anti-dandruff shampoos. Administering these agents systemically at the micromolar concentrations evaluated here (1–10 µM) introduces risks of cytotoxicity to normal tissues. Because our screening focused exclusively on malignant LUSC cell lines without evaluating their impact on healthy cells or tissues, these raw biological hits require follow-up studies to determine their suitability for clinically viable systemic candidates. In contrast, while 2-methoxyestradiol possesses more advanced in vivo evaluations in other cancer types, its specific pharmacokinetic properties, drug stability, and delivery feasibility within the context of LUSC patients will require further validation.

Importantly, these limitations reflect the deliberate parameters of our study design rather than methodological oversights. This work was conceptualized strictly as a high-throughput, foundational screening phase intended to efficiently narrow down a vast chemical library into high-priority biological candidates using well-characterized model systems of LUSCs. By establishing this essential baseline of selective 3D efficacy, we have provided several top compounds while avoiding the premature and resource-intensive use of animal models. To progress beyond this baseline and elevate the translational relevance of these findings, future work must transition these candidates into advanced co-cultures and patient-derived organoid systems to better replicate human TME physiology. Subsequent evaluation should prioritize expanding testing across multiple LUSC cell lines alongside normal lung epithelial cells to define clear therapeutic windows. Finally, extensive pharmacodynamic and pharmacokinetic (PK/PD) profiling in vivo will be required to rigorously assess systemic delivery, metabolic stability, and the precise molecular pathways governing cell death in vivo.

## Conclusions

Using a high-throughput drug screening strategy, we have identified several drugs with newly defined anti-cancer activity as a result of their ability to both kill cells growing in a 2D model system and reduce spheroid growth in a 3D model system. The drug 2-methoxyestradiol has emerged as the top candidate inhibiting spheroid growth and reducing cell viability, with stronger effects in more metastatic cell models. These results suggest that the anti-cancer efficacy of these newly identified compounds, particularly 2-methoxyestradiol, should be prioritized in future preclinical and clinical trials for LUSC.

## Supporting information

S1 Fig Drugs cytotoxic towards both NCI-H2170 and NCI-H2170 +HA-CREB3L1 LUSC cells. NCI-H2170 (circles; metastatic) and NCI-H2170 +HA-CREB3L1 LUSC (squares; poorly metastatic) cells were treated with the indicated drugs over a range of concentrations up to 10 µM as detailed in the methods. The number of live cells in the drug treated samples was quantified and compared to total cells in vehicle control wells to determine cell viability at each drug concentration. These drugs were highly cytotoxic at all concentrations tested (down to 50 nM).
(PDF)

S2 Fig Drugs ineffective or poorly effective towards both NCI-H2170 and NCI-H2170 +HA-CREB3L1 LUSC cells. NCI-H2170 (circles; metastatic) and NCI-H2170 +HA-CREB3L1 LUSC (squares; poorly metastatic) cells were treated with the indicated drugs over a range of concentrations up to 10 µM as detailed in the methods. The number of live cells in the drug treated samples was quantified and compared to total cells in vehicle control wells to determine cell viability at each drug concentration. **A**) These drugs had little or no effect on cell viability. **B**) These drugs were effective at concentrations ≥10 µM.
(PDF)

S3 Fig Drugs equally or less effective for metastatic NCI-H2170 LUSC cells as compared to their less metastatic CREB3L1-expressing counterparts. The indicated cells were grown as 3D spheroids and treated with drug (10 µM) for 4 days. The volume of each spheroid was measured to quantify the impact of drug treatment compared to the DMSO control treated samples. The mean ± SEM from 4 biological replicates from each condition.
(PDF)

S4 Fig Reduced drug concentrations (1 µM) are not effective against NCI-H2170 cells grown in 3D spheroids. **A)** NCI-H2170 cells were grown as 3D spheroids and treated with drug (1 µM) for 4 days. The volume of each spheroid was measured to quantify the impact of drug treatment compared to the DMSO control treated samples. Mean ±SEM from 4 independent spheroids. A t-test was used to assess statistically significant differences. These drugs showed no significant differences between treated (1 µM) and untreated DMSO control samples.
(PDF)

S1 Table Selleckchem L1300 FDA-approved drug library compounds and properties. Details of each drug in the library (2,580) with their properties.
(XLSX)

S2 Table Drugs most cytotoxic towards NCI-H2170. Drug treatments (10 µM) for 4 days giving rise to <20% viability for NCI-H2170 cells are shown.
(XLSX)

S3 Table Drugs showing the largest reduction in spheroid size for NCI-H2170 cells. Drug treatments (10 µM) for 4 days giving rise to <50% volume for NCI-H2170 cells are shown.
(XLSX)
