# Supplementary material for: 2-methoxyestradiol is effective in 2D and 3D models of NCI-H2170 lung squamous cell carcinoma cells
Source: PLoS One. 2026 Aug 4;21(8):e0355486. doi: 10.1371/journal.pone.0355486 (PMC13436739; doi:10.1371/journal.pone.0355486)

A Ineffective drugs

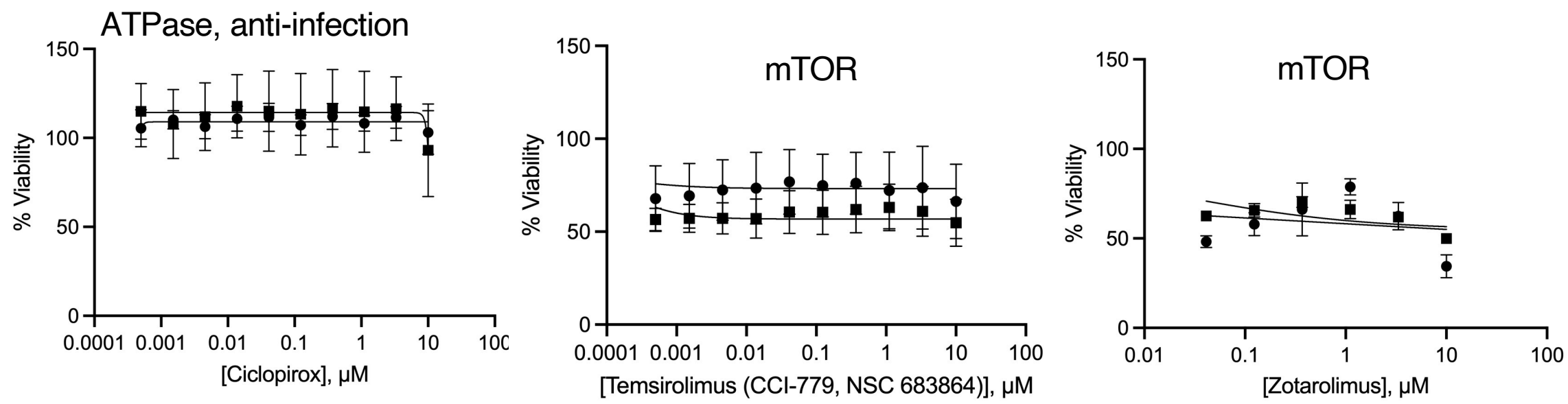

B Drug requiring high concentrations (>10 μM)

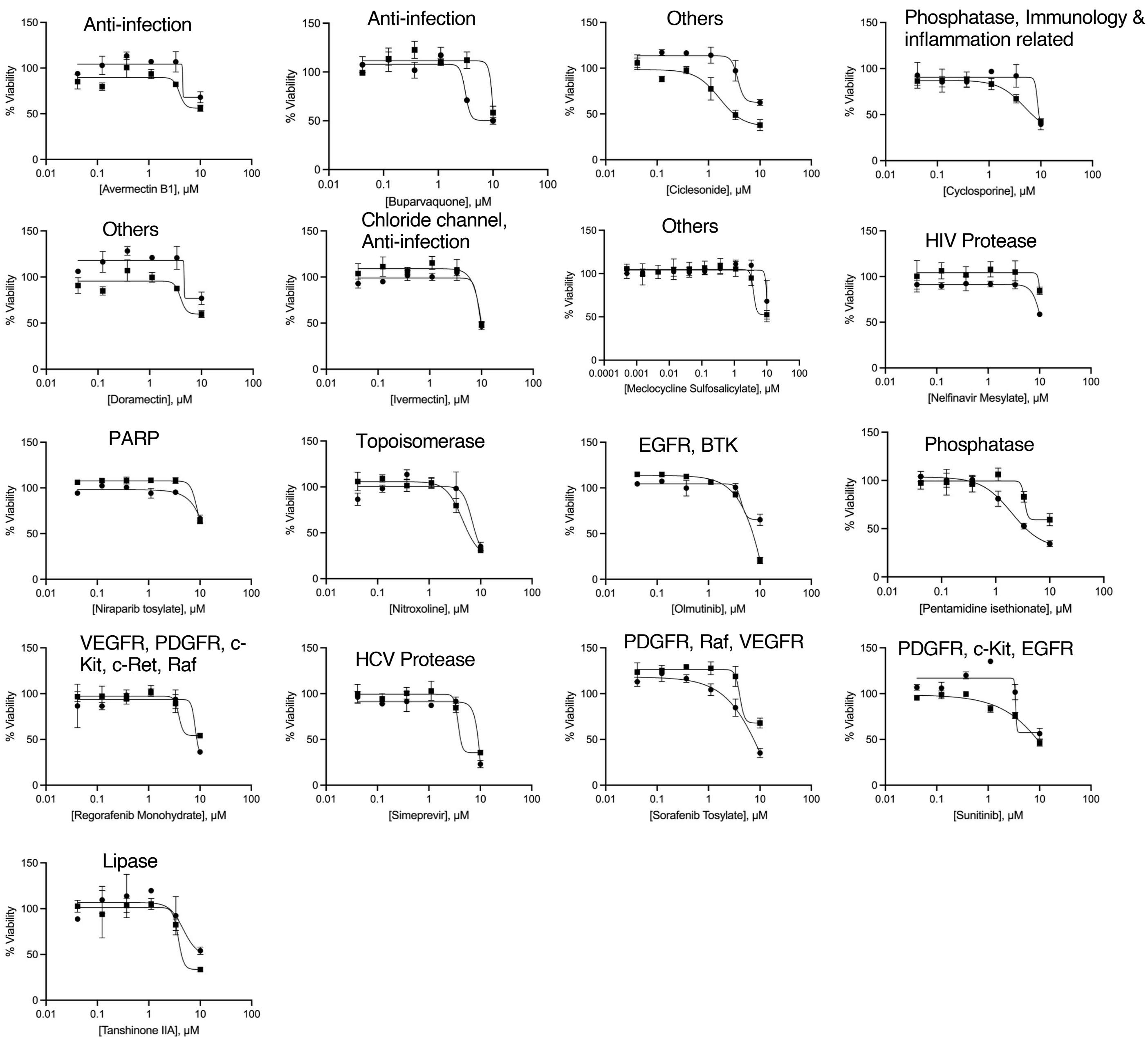

Supplement: Supplementary file 2 [file pone.0355486.s002.pdf]
