# Supplementary material for: 2-methoxyestradiol is effective in 2D and 3D models of NCI-H2170 lung squamous cell carcinoma cells
Source: PLoS One. 2026 Aug 4;21(8):e0355486. doi: 10.1371/journal.pone.0355486 (PMC13436739; doi:10.1371/journal.pone.0355486)

Mellor, S3 Fig

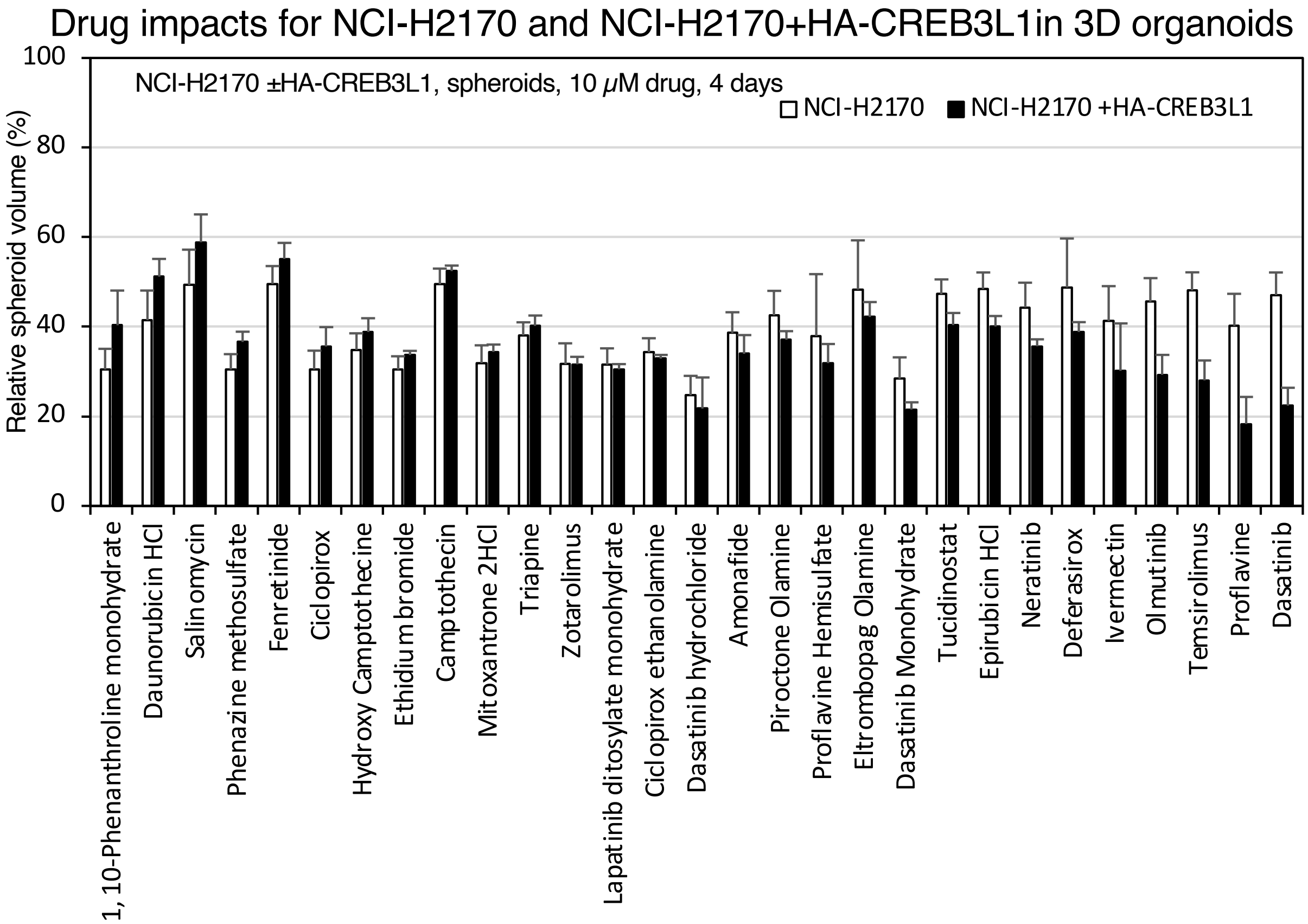

Supplement: Supplementary file 3 [file pone.0355486.s003.pdf]
