# Supplementary material for: 2-methoxyestradiol is effective in 2D and 3D models of NCI-H2170 lung squamous cell carcinoma cells
Source: PLoS One. 2026 Aug 4;21(8):e0355486. doi: 10.1371/journal.pone.0355486 (PMC13436739; doi:10.1371/journal.pone.0355486)

Ineffective drugs for 3D organoids of NCI-H2170 cells

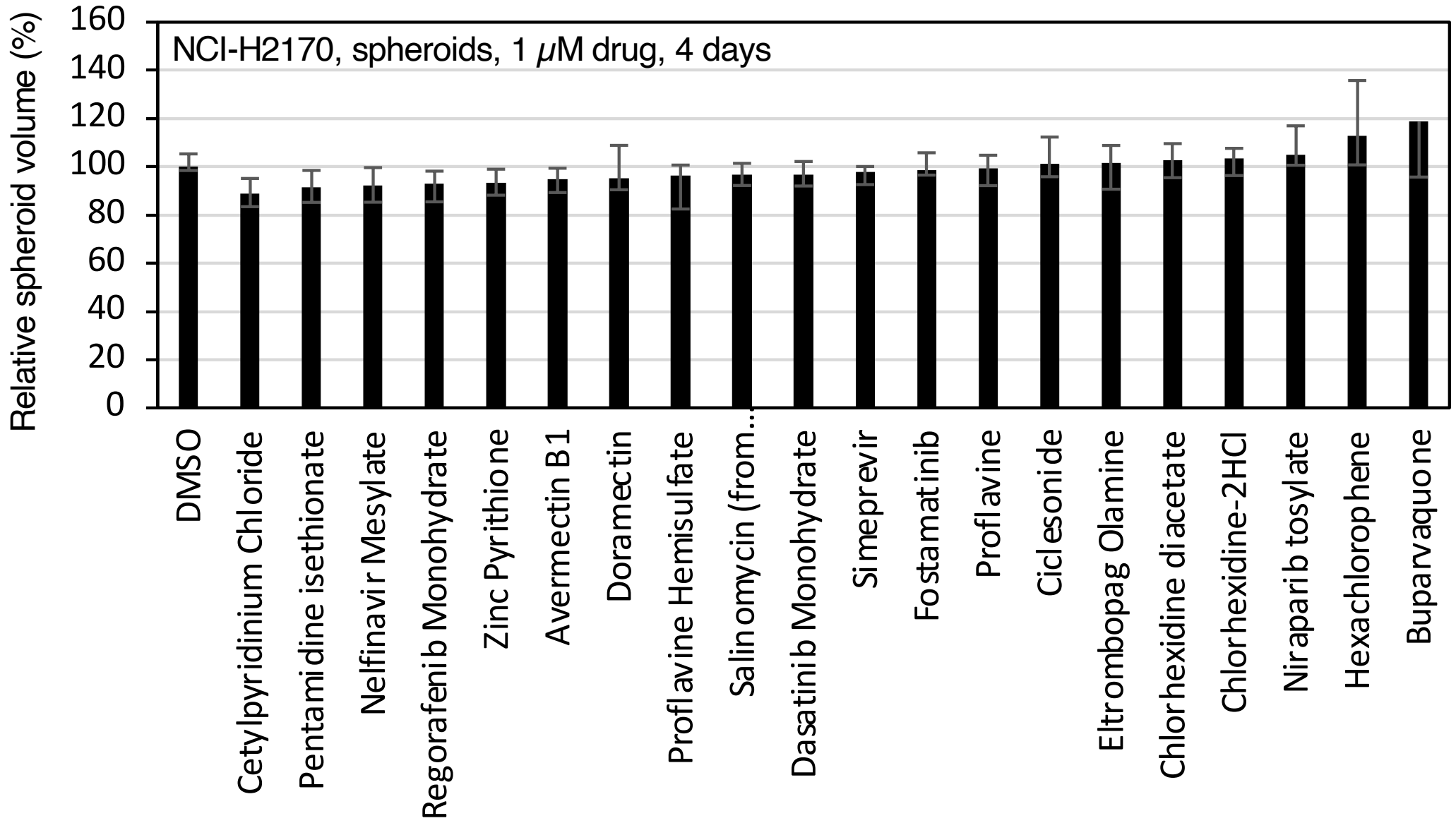

Supplement: Supplementary file 4 [file pone.0355486.s004.pdf]
